# Come! What Say You?

**Published:** 1879-07

**Authors:** Thos. K. Beecher


### COME! WHAT SAY YOU?

THOS. K. BEECHER.

"If you will be the bringer of new doctrines, do not look for a welcome. Walter Bagehot concludes that to the ordinary mind no effort is so painful as the reception of a new idea; and Leslie Stephen says: "Mankind resent nothing so much as the intrusion upon them of a new and disturbing truth.'"

Just see, for instance, what men say and do (or don't do) in the matter of the cremation of the dead.

One class, the emotional, turn off the whole thing with a shrug and a shiver. "What a horrible idea! I can't bear to think of it;"—neither will they think of the alternative—burial and slow decay. By such, to think at all upon unpleasant topics is shunned.

Another class, the superstitious, suppose themselves to "believe in the resurrection of the body" so stoutly, that to burn it is sacrilege. Let the dead be laid feet to the east, that they may face the resurrection sunrise. But they never quote St. Paul's words—it is sown a natural body, it is raised *a spiritual body*. There is a natural body, and there is a spiritual body.

Another class, are the conservative Chinese-like folks, who shrink from doing any new thing. We always have buried the dead, and so we may as well keep on burying them. Don't bother us with your new-fangled scientific notions. These valuable citizens abide in their steadfastness until a pestilence or a red-hot fight wakes them up, and then they usually decide aright.

Loving sentiments inspire another class. They crave a chance to lavish treasure upon their loved ones dead. Costly woods for the coffins, satin and silver or gold for the "trimmings," a high-priced lot and monument. It relieves a loving heart to lavish treasure upon the memory of the beloved.

All these classes are inaccessible to the appeals of the advocates of cremation. They do not even resist. They neglect the whole matter. What is needed is, therefore, not talk but action, QUIET, manful action.

Let any twenty citizens keep out of the newspapers, and build a neat and convenient crematory, and when the sad necessity arises go quietly, with no parade, and DO the proposed thing, and then stand by. Time will educate the masses, and win all intelligent people to the side of neatness, health, and good taste.

The writer of these lines has received countless expressions of approval, but only one *bona fide* subscription of stock. Except in a quiet and confidential way he is not willing to receive such subscriptions. But he insists, that no man can offer a valid objection to the proposed reform of our mortuary usages; nor can any reasonable man defend them as they are.

He who has read of Pere le Chaise in Paris, or of water burial in New Orleans, cannot defend old and populous cemeteries on the score of decency.

He who has aided in removing the remains of the dead from one to another grave, will not easily reconcile himself to burials ever after.

He who knows that dust returns to dust, and the soul to God, (no matter what disposal we make of the dead) can not hesitate long whether a short and sweet road, or a long and nasty one is preferable.

He who knows aught of exhalations in the air, and leechings through the soil from the festering dead, will not, cannot long hesitate to say that some change for the better is demanded.

He who has seen old graveyards secularized, and broken bones, skulls, and coffin-splinters carted away and dumped, will not plead that burial is safe and sacred!

He who has read of the great merchant Stewart, and of the generous Dwight, and of their post mortem adventures with body-snatchers, coroners, doctors and insurance men, is satisfied that cremation had been better. Then, indeed, the wicked had ceased from troubling, and the wealthy had been at rest.

And surely a beautiful monument is not made more beautifnl by setting its deep foundations on a lap of mould and decay!

Peace to the ashes of all the dead, say we all. Why not say, peace and purity be with our dead? Except for the clean aud cleansing agency of fire, our language had never gained the beautiful words *pure* and *purity*.

The writer is ripe and ready to act. He invites manly, intelligent, courageous, BUT QUIET co-operation. Let every man be clearly persuaded in his own mind, and so let him ACT.

